# A simple and rapid assay of lysosomal-targeting CDy6 for long-term real-time viability assessments in 2D and 3D in vitro culture models

**DOI:** 10.1038/s41598-023-49844-1

**Published:** 2023-12-27

**Authors:** Chanhan Kang, Won-Soo Yun, Yun-Mi Jeong

**Affiliations:** Department of Mechanical Engineering, Tech University of Korea, 237 Sangidaehak Street, Si-Heung City, Republic of Korea

**Keywords:** Biochemistry, Chemical biology, Molecular biology, Medical research, Molecular medicine

## Abstract

CDy6, a BODIPY-derived compound, is used to label lysosomes and visualize proliferating cells. However, its effectiveness in long-term, real-time cell viability assays using 2D or 3D cell culture models is unclear. We evaluated the suitability of CDy6 by assessing cell health using human keratinocyte and fibroblast cell lines in both models. Cells were stained with CDy6 or other dyes and fluorescent images were obtained with confocal microscopy. CLV extracts derived from CDy6-stained HaCaT cells were also dissolved with DMSO and analyzed using a spectrometer. Furthermore, we added CDy6-stained collagen hydrogels to CCD-986sk cells, loaded them into a frame construction to establish a 3D dermal layer for long-term culture, and analyzed the status of the CLVs. The CLV method, also measured using a spectrometer, yielded results similar to MTT assay for validating viability. In contrast to calcein AM staining, the CLV method allows for both absorbance measurement and imaging under short-term and long-term culture conditions with less cytotoxicity. In conclusion, the CLV method provides a simple and sensitive tool for assessing the status of live cells in 2D and 3D in vitro cell culture models and can be used as an alternative to animal testing, such as with 3D artificial skin models.

## Introduction

In vitro cell-based assays using 2D and 3D culture systems are advantageous in drug discovery and drug repositioning due to their ability to overcome various challenges such as drug uptake, intracellular stability, cellular function maintenance, cytotoxicity, and cell survival^1,2^. These assays have become essential in evaluating the efficacy and safety of novel drugs in vitro^1,2^. One widely used method is the MTT (3-(4,5-dimethylthiazol-2-yl)-2,5-diphenyltetrazolium bromide) assay, which quantifies cell viability, proliferation, and cytotoxicity based on the reduction of MTT to formazan by mitochondrial dehydrogenase enzymes in viable cells^3–5^. The purple formazan product is solubilized in DMSO and measured by absorbance at 570 nm^3–5^. Although the MTT assay remains a popular choice due to its simplicity, reliability, and cost-effectiveness, it still has some substantial limitations, including challenges in generating accurate qualitative analyses. For example, MTT dye primarily absorbs light in the range of 570–600 nm, which differs from the wavelength range commonly used for many fluorescent dyes, making it undetectable using microscopy^5^. Moreover, the measurement of mitochondrial dehydrogenase enzyme activity targeted by MTT assays and analyzed with spectrometers can vary among cell types and experimental conditions. In particular, the seeding cell number, the concentration of MTT reagent added to the cells, the incubation time with MTT, the type of culture media, the removal of cells’ supernatant flowing MTT incubation, the wavelength at which optical density is measured, and the tested treatment all impact the accuracy of qualitative analysis. These confounding factors can potentially result in cell death or toxicity prior to the assay measurements and also lead to misinterpretation of the assay results^5^. This highlights the importance of employing rigorous validation methods and control conditions when utilizing the MTT assay to ensure the reliable interpretation of cell viability data. Nonetheless, while careful experimental design and proper controls can yield accurate results using the MTT assay, there is still a need to develop additional validation methods that can complement the MTT assay to provide a more comprehensive assessment of cell health.

Calcein AM is a well-established hydrophobic fluorescent dye that is widely used to assess cell viability and cellular processes in vitro and in vivo^6,7^. This dye can easily penetrate cell membranes and is converted into a highly fluorescent compound, calcein, by intracellular esterases. Calcein AM is generally used as a calcium indicator in biological systems, and many researchers have utilized it to stain live cells with very visible, green fluorescence^6–9^. For instance, a previous study developed an imaging-based platform that uses calcein AM to predict individual radioresponse, enable real-time monitoring of tumor growth, and evaluate responsiveness to therapy^8^. Another study demonstrated that calcein AM can serve as a sensitive detector of intracellular oxidative activity, with useful applications for real-time imaging in confocal microscopy^9^. However, calcium-targeted fluorescent probes like calcein AM have several drawbacks when used to stain animal or human cells due to potential toxicity and limited precision, leading to potential interference from other ions or molecules in the cellular environment.

CDy6 is a BODIPY (boron-dipyrromethene)-derived compound used as a long-term, real-time, and photostable marker to visualize lysosomal vesicles, which are membranous organelles that are found in most mammalian cell types and play important roles in cell biology and function^10^. Although CDy6-mediated labeling has been shown to be a useful method for monitoring lysosome dynamics, it is unclear whether CDy6 can be used to evaluate cell health, i.e. cell viability and proliferation, in 2D and 3D in vitro cell culture models under long-term and real-time conditions. This study aimed to develop a simple and effective one-step assay for evaluating cell status via CDy6-targeted lysosomal vesicles (CLVs) in 2D and 3D in vitro cell culture models. The assay was designed to provide quantitative and qualitative analysis of cell and organismal homeostasis based on CLVs derived from dynamic lysosomes using both a spectrometer and microscope.

## Results

### CLV-DMSO extraction and measurement of CDy6 intensity in the short-term culture condition

**Figure 1:**
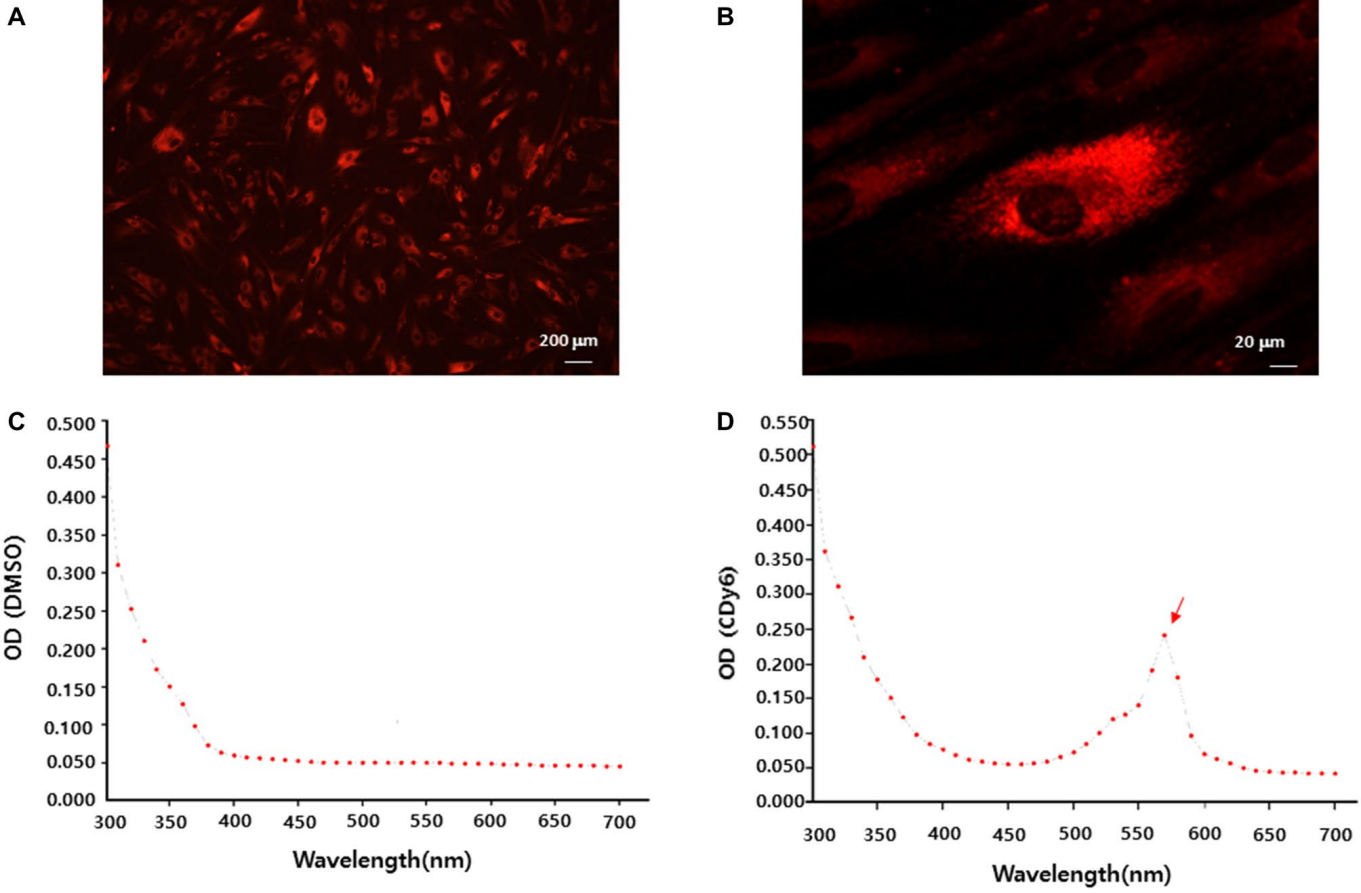
CLV-DMSO extraction from CDy6-stained HaCaT cells can measure intercellular lysosomal vesicle levels at an absorbance spectrum of 570 nm. (**A**,**B**) Fluorescence live-cell imaging of CDy6-labeled HaCaT cells after CDy6 staining (100 ng/mL) for 1 h. The specificity and patterns of the staining very visibly display the form of the lysosomal vesicles in the cytoplasm. Scale bars, 200 µm (**A**) and 20 µm (**B**). (**C**,**D**) The absorbance spectra of the DMSO only and the CLV-DMSO extract from CDy6-stained HaCaT cells were measured with an ELISA reader. DMSO is used as a negative control. The red arrow indicates the peak excitation/emission wavelengths of CDy6, reaching 570/585 nm.

CDy6 is a fluorescent dye that specifically labels lysosomal vesicles in live cells^10^. It has been shown to be a reliable tool for tracking lysosomal dynamics in live cells over time, given its excellent photostability in vitro and in vivo^10^. In addition to its stability and specificity for lysosomes, CDy6 also exhibits low toxicity and does not affect normal cellular processes^10^. Based on these findings, we investigated whether CLVs could be extracted from live cells using DMSO, with the fluorescence intensity of the CLV-DMSO extracts then measured to assess cell viability in 2D culture models. To test this, HaCaT cells were stained with CDy6 for 1 h, after which the conditioned medium was removed and the CLVs in live cells were incubated with DMSO for 1 h at room temperature (RT). The fluorescent absorbance of the CLVs was then measured using a spectrometer scanning between 300 and 700 nm. The results show that CLVs were widely distributed in the cytoplasm of live cells (Fig. 1A,B). CLV-DMSO extracts derived from HaCaT cells exhibited a sharper peak in CDy6 excitation/emission wavelengths, reaching 570/585 nm, compared to DMSO alone (Fig. 1C,D). These wavelengths were equivalent to the wavelengths of CDy6 alone, indicating that the extracted CLVs were detectable using a spectrometer, in line with our previously published study^10^ (Fig. S1). These observations indicate that this method of isolating and measuring CLVs using DMSO extraction and spectrometry might be useful for assessing cell viability.

### Comparison of absorbance evaluated by calcein AM and CLV-DMSO extraction assays

**Figure 2:**
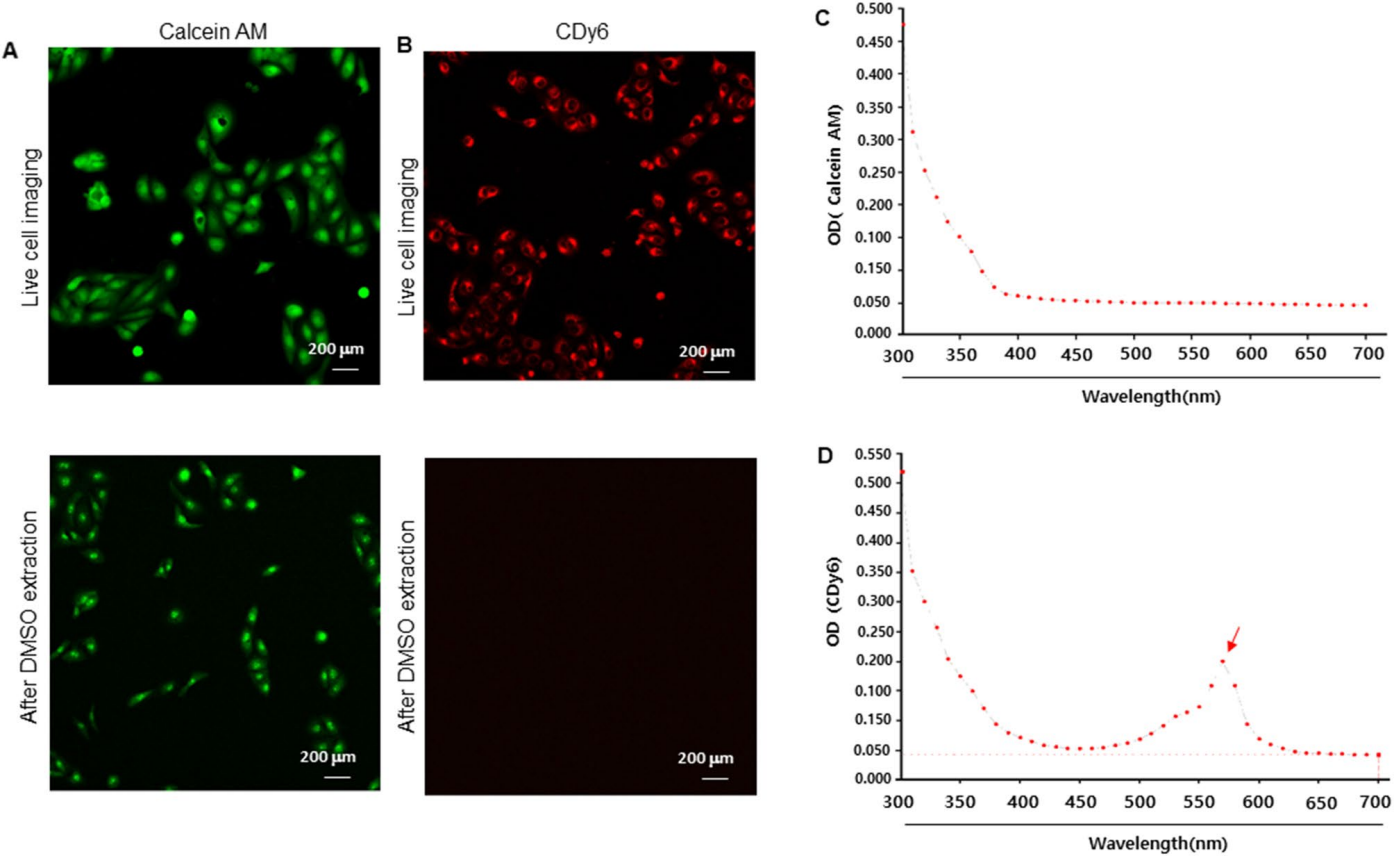
Comparative assessment between CDy6-stained and calcein AM-stained HaCaT cells before and after DMSO extraction. (**A**,**B**) After staining with calcein AM (100 nM) and CDy6 (100 ng/mL), fluorescence images of live cells were obtained using a confocal microscope at the indicated time points as described in more detail in the materials and methods section. (**C**,**D**) The absorbance spectrum of the CLV-DMSO and calcein AM-DMSO extracts from each group was measured with an ELISA reader. The red arrow indicates the peak excitation/emission wavelengths of CDy6, reaching 570/585 nm.

To investigate the potential use of CLVs for assessing cell viability and proliferation, we compared the images and the fluorescence intensity of CDy6 and calcein AM. HaCaT cells were stained with either calcein AM or CDy6 for 1 h, removed from the conditioned media, and incubated with DMSO for 1 h at RT. The fluorescence intensity of both dyes was measured using a spectrometer scanning from 300 to 700 nm. The live cell images were acquired with a confocal microscope. As shown in Fig. 2A,B, the confocal images display the calcein AM-stained and CDy6-stained HaCaT cells before and after DMSO extraction. After DMSO extraction, calcein-stained HaCaT cells clearly remain (Fig. 2A). In contrast, after DMSO extraction, CLVs were not observed in the CDy6-stained HaCaT cells, indicating thorough extraction (Fig. 2B). Consistent with these findings, absorbance spectrum analysis detected no fluctuation in fluorescence intensity of the calcein AM dye (Fig. 2C) after DMSO extraction, while CLV-DMSO extracts revealed a sharp increase in fluorescence intensity in the presence of CDy6 at 570 nm/580 nm (Fig. 2D). These results indicate that CDy6 can serve to assess the viability of live cells using both absorbance analysis and live cell imaging in the short term.

### Comparison of cell proliferation in long-term culture conditions using calcein AM and CDy6 staining

**Figure 3:**
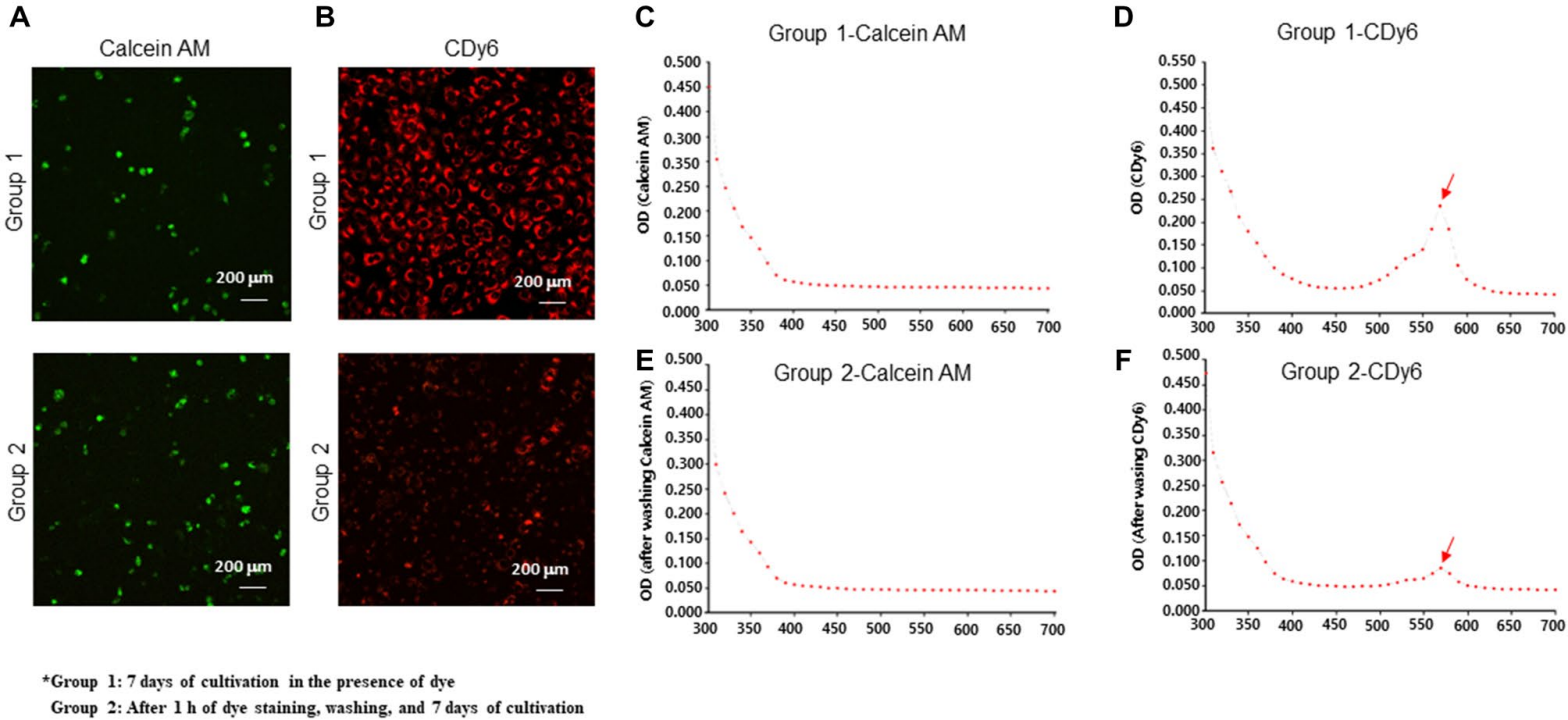
Comparative analysis of CDy6 and calcein AM staining in HaCaT cells before and after washing. (**A**,**B**) Live cell fluorescence images were obtained using a confocal microscope at specified time points after staining HaCaT cells with CDy6 (100 ng/mL) and calcein AM (100 nM) with and without washing, as described in detail in the Materials and methods section as well as in Fig. S2 in the Supplementary information. (**C**–**F**) Absorbance spectra of the CLV-DMSO and calcein AM-DMSO extracts were measured with an ELISA reader before and after washing each sample. The red arrow indicates the peak excitation/emission wavelengths of CDy6, reaching 570/585 nm. Group 1 includes samples that underwent 7 days of cultivation in the presence of each dye. Group 2 includes samples stained with each dye for 1 h, followed by washing, and 7 days of cultivation.

**Figure 4:**
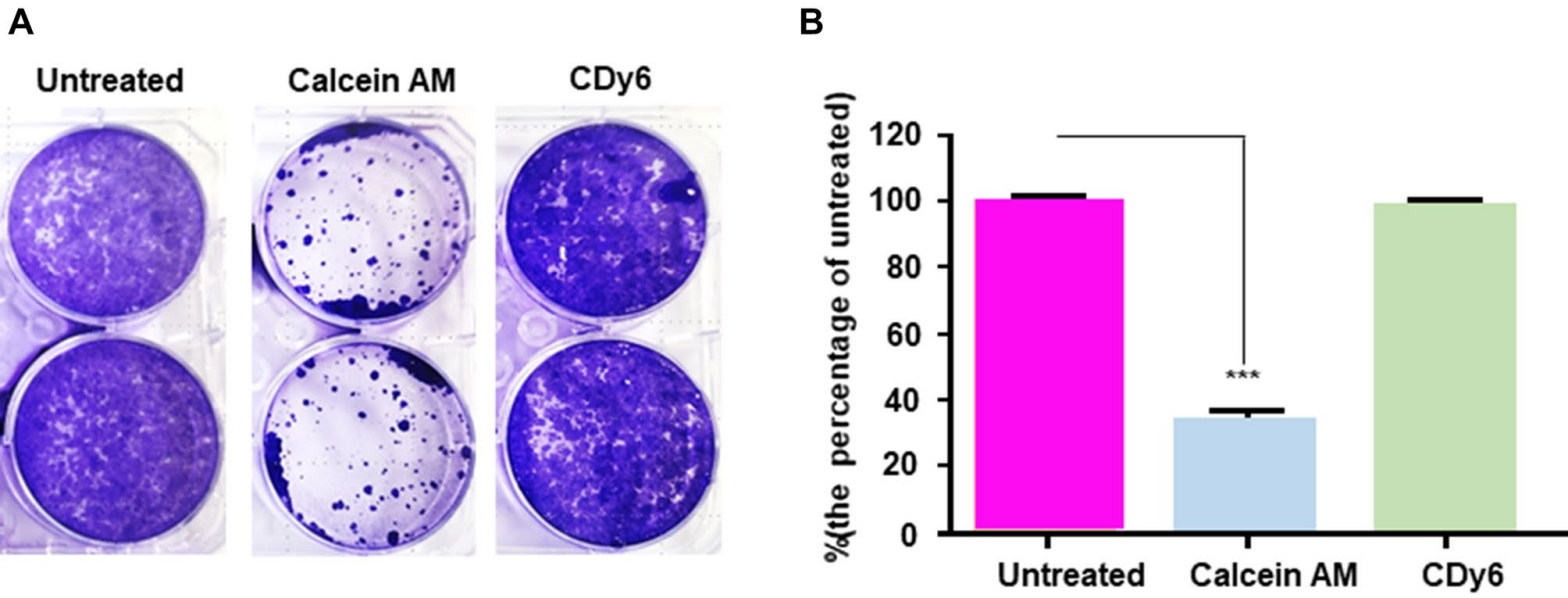
Effect of CDy6 and Calcein AM staining on cell viability in HaCaT cells under the long-term culture condition. (**A**) HaCaT cells were stained with CDy6 (100 ng/mL) or Calcein AM (100 nM) for 7 days. Cell viability was assessed by crystal violet staining. Representative images of crystal violet staining after 7 days are shown. (**B**) Bar graph showing cell proliferation in each group: untreated (pink), calcein AM (blue), and CDy6 (green). Data represent the mean ± SD from three independent experiments performed in triplicate as a percentage of the untreated group. Data was analyzed using Student’s t-test. ***P < 0.001 versus corresponding controls.

**Figure 5:**
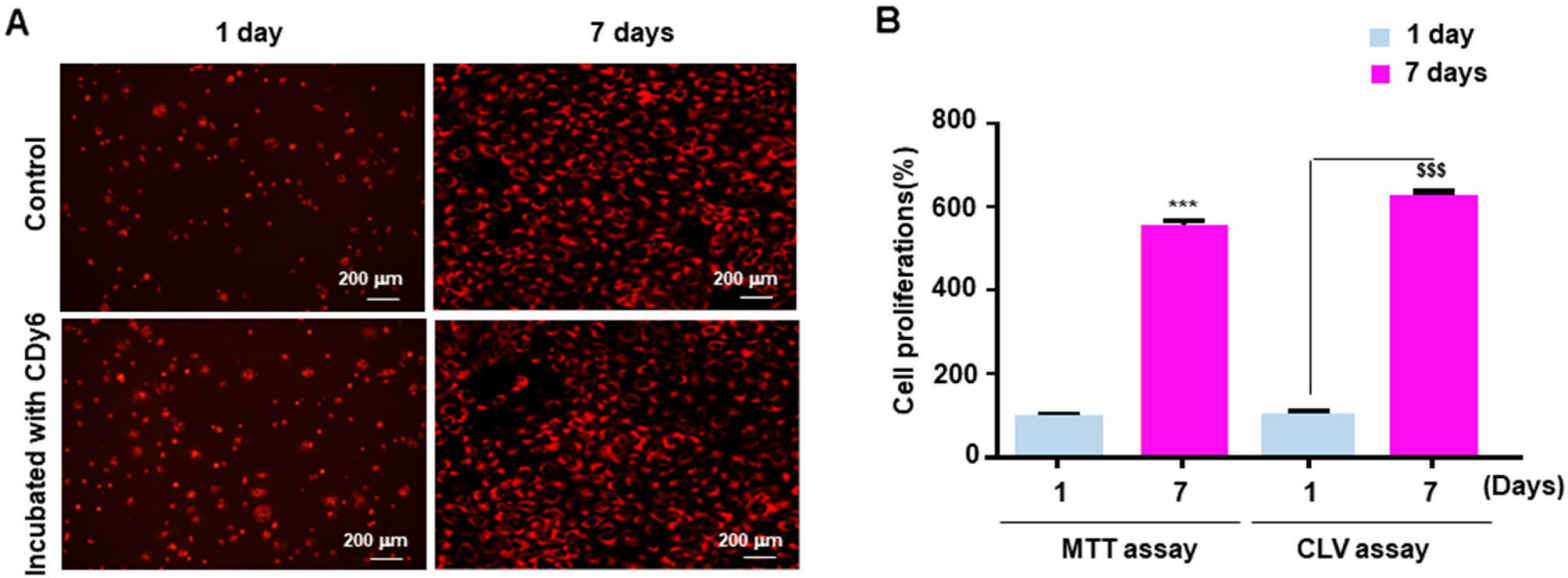
Comparison of MTT assay and CDy6 staining for monitoring long-term culture of HaCaT cells. (**A**) HaCaT cells were stained with CDy6 (100 ng/mL) and cultured for 7 days, and live cell fluorescence images were obtained using a confocal microscope, as described in the Materials and methods section. (**B**) The absorbance spectra of the CLV-DMSO and MTT assay products were measured with an ELISA reader for each group (blue, 1 days incubation; pink, 7 days incubation). Data represent the mean ± SD of from three independent experiments performed in triplicate as a percentage of the 1 days. Data was analyzed using Student’s *t*-test. ***or $$$ P < 0.001 versus corresponding controls.

To further examine the effectiveness of the calcein AM and CDy6 staining methods for analyzing cell proliferation in real-time over long-term culture conditions, we performed two sets of experiments. In one experiment, HaCaT cells were incubated continuously in the presence of each dye during long-term culture conditions. In the other experiment, after incubation in each dye for 1 h, the cells were also washed and then incubated again during long-term culture conditions (Fig. S2). Confocal images showed that most of the unwashed calcein-AM-labeled HaCaT cells (group 1) and the washed calcein-AM-labeled HaCaT cells (group 2) were dead or displayed inhibited cell proliferation, whereas the proliferation of the HaCaT cells (in both group 1 and group 2) were not affected in the presence or absence of CLVs, as previously described^10^ (Fig. 3A,B). These results are consistent with the fluorescence intensity graph of CLV-DMSO extracts using a spectrometer scanning between 300 and 700 nm (Fig. 3C–F). After the washing step performed on group 2, the wavelength intensity of CDy6 in the CLV-DMSO extract was lower than that of group 1 after 7 days when measured with a spectrometer. These observations suggest a novel one-step method for determining both qualitative and quantitative analyses of cell viability and cell proliferation for short and long-term conditions via lysosomal targeted CDy6 staining. In addition, we examined whether CLVs could enable long-term and real-time tracking of live cells using time-lapse imaging. This ability was confirmed through the stable photo intensity exhibited under long-term real-time conditions with live cells (as shown, for example, at 24 h in Movie S1 and 92 h in Movie S2). To further validate our findings, we stained HaCaT cells with CDy6 for 7 days. Crystal violet staining assays and confocal images demonstrated that the CLVs of these cells remained healthy as compared to the control under both short-term and long-term conditions, whereas calcein AM negatively impacted cell proliferation (Figs. 4 and 5). Moreover, quantitative analysis of the CLV-DMSO extracts showed similar results in measuring cell viability and cell proliferation as those obtained with the MTT assay when analyzed with a spectrometer (Fig. 5B). These findings suggest that the CLV method can serve as a robust tool for real-time monitoring of cell health in live cells using absorbance analysis and confocal microscopy.

### CLVs enable the monitoring of live cells in 3D in vitro cell culture models, facilitating the assessment of cell health in live cells

**Figure 6:**
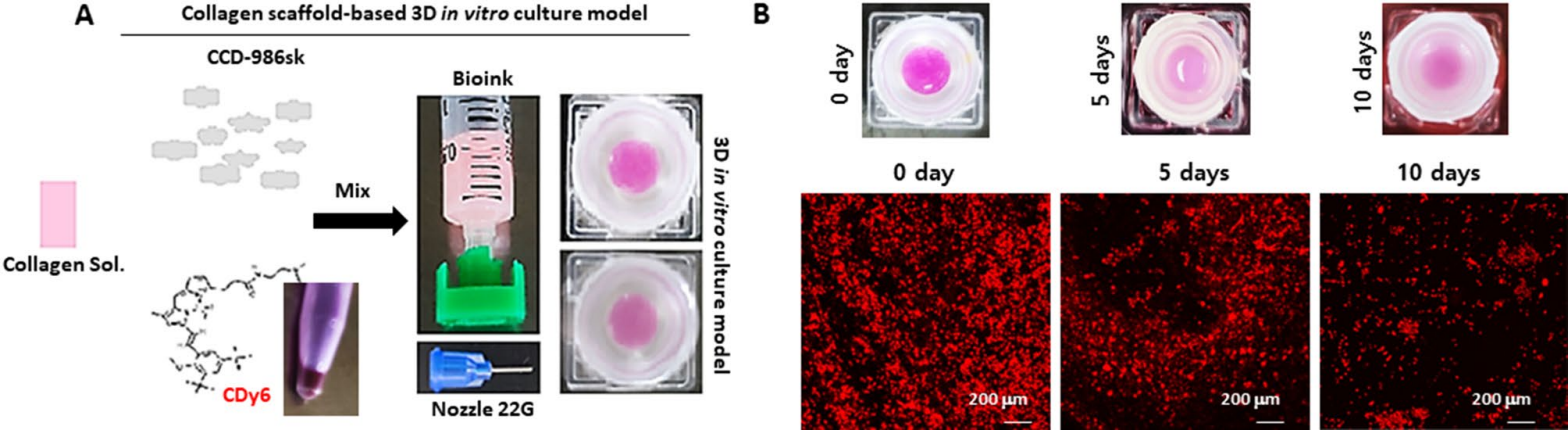
Real-time monitoring of CDy6-stained 3D dermal layer using CLVs. (**A**) Schematic representation of the biofabrication process for generating the CDy6-labeled 3D dermal layer in the 3D in vitro cell culture model, as described in the Materials and Methods section. (**B**) Confocal live imaging of the 3D dermal layer with CLVs at the indicated time points, demonstrating real-time visualization of the cell status. Scale bars, 200 µm.

**Figure 7:**
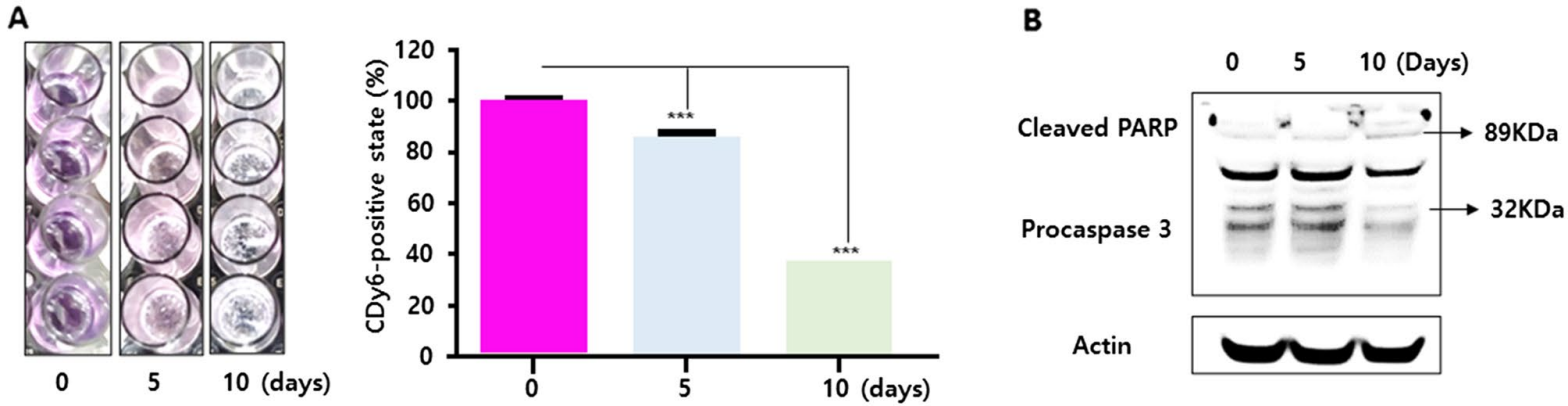
Analysis of the CDy6-stained 3D dermal layer using CLVs and Western blot. (A) CLV-DMSO extracts were obtained from CDy6-stained 3D dermal layers at the indicated time points, and the living cells were quantified using an ELISA reader. Representative images of the CLV-DMSO extracts are shown. Data represent the mean ± SD from three independent experiments performed in triplicate as a percentage of the untreated group. Data was analyzed using Student’s *t*-test. ***P < 0.001 versus corresponding controls. (B) Western blot analysis of caspase expression in each group, with actin as a loading control.

In recent years, researchers have been developing 3D in vitro cell culture models that more accurately represent the complex cellular microenvironment of human tissues^1,2,11^. These advanced technologies have enabled researchers to study cellular behavior in a more realistic context, providing valuable insights into disease mechanisms and drug development ^1,2,11^. H&E staining is a commonly used method for evaluating the effectiveness of drugs in 3D in vitro cell culture models^12^. However, these methods have some limitations, such as the complexity of their protocols and the lengthy time required for analyzing samples. Lysosome transport could instead serve as a marker to more efficiently assess cell viability in 3D in vitro cell culture models under real-time and long-term conditions. Lysosomal transport is known to be essential to cell health^13^. Dysfunctional lysosomes can lead to an accumulation of waste materials within cells that has been associated with various diseases, such as lysosomal storage disease and cancer^13–15^. Research on the connection between lysosomes and cell health is actively pursued in fields such as cell biology, biochemistry, and immunology, providing insights into maintaining cell health and preventing diseases. To address these issues, we explored the potential of using CLVs to assess the health of cells in 3D in vitro cell culture models, such as in a 3D artificial skin model. We generated a 3D dermal layer for a skin equivalent model using a collagen-CDy6 mixed hydrogel and a CCD-986sk cell line, which is a human fibroblast cell line (Fig. 6A). The typical culture period for 3D dermal layers in vitro is between 7 and 10 days. The CCD-986sk cells were mixed with the collagen-CDy6-mixed hydrogel solution and loaded into a frame construction. This was followed by culturing the construct in a complete medium under long-term culture conditions. During this period, the culture was monitored regularly to assess the stability and detectability of the CLV-labeled live cells using both absorbance analysis and confocal microscopy to visualize the cells and evaluate their status. The results indicate that the live cells labeled with CLVs remained stable and detectable throughout the culture period, suggesting that the method is effective for assessing the health of the cells in 3D in vitro cell culture models, such as a 3D artificial skin model (Fig. 6B). Furthermore, to confirm the reliability of our observations, we performed western blot analysis on the lysates of the cells. It is well-known that poly-ADP-ribose polymerase (PARP) cleavage and procaspase-3 downregulation are hallmarks of apoptosis^16^. As depicted in Fig. 7B, after 10 days incubation of the 3D dermal layer in the skin equivalent model, we observed cleaved PARP and a decrease in procaspase-3 at the protein level. The results of the western blot analysis are consistent with the absorbance analysis and imaging, indicating that the CLV extract provides a reliable means for monitoring cell status in 3D in vitro cell culture models (Fig. 7). Therefore, our findings demonstrate that CLVs could be used for studying cell health in both 2D and 3D in vitro cell culture models, potentially leading to the development of new alternative methods for drug efficacy and safety assessment, replacing the need for animal testing.

## Discussion

The present study highlights a simple and rapid assay that utilizes CLVs to assess cell status in 2D and 3D in vitro cell culture models under short- and long-term culture conditions. Our method involves staining cells with CDy6, extracting the CLVs using DMSO, measuring the fluorescence intensity of CLV-DMSO extracts using a spectrophotometer, and capturing live cell imaging using confocal microscopy. Our findings show that this CLV-mediated assay can more effectively assess cell viability and proliferation in 2D culture conditions compared to the commonly used MTT assay. Moreover, CLVs were stable and detectable in 3D in vitro cell culture models using a spectrometer, thereby validating the use of CLVs for real-time monitoring of cell health with absorbance and confocal microscopy analysis. In general, cell viability assays are categorized into various types based on the method of analysis used. Dye exclusion assays, colorimetric assays, fluorometric assays, luminometric assays, and flow cytometric assays are commonly used methods for assessing cell viability. A previous report discussed the importance of reliable and efficient assays for assessing cell viability and proliferation in 3D culture models while avoiding interference with the test compound^1,11^. The authors suggested that some assays may not be suitable for 3D in vitro cell culture models due to limitations in penetration and sensitivity, and emphasized the need for carefully selecting and validating cell viability assays for use in such models^1,11^. In comparison to current dyes, the simple and rapid CLV-mediated assay can serve as a valuable tool for assessing cell health in 2D and 3D in vitro cell culture models and have the potential to compensate for the limitations of current dyes.

Our results demonstrate how the proposed CLV-mediated assay can effectively track lysosomal transport in live cells. A live cell exhibits the complex transport of lysosomes, which is essential for maintaining cellular homeostasis and is involved in multiple cellular processes^14,17,18^. Research on the connection between lysosomes and cell health is actively pursued in fields such as cell biology, biochemistry, and immunology, providing insight into maintaining cell health^14,17,18^. In contrast, cell death occurs through three main morphologically distinct processes: necrosis, apoptosis, and autophagic cell death. Necrosis is characterized by organelle swelling, rupture of the plasma membrane, and disintegration of cellular structures, while more orderly apoptosis is marked by cellular shrinkage, nuclear fragmentation, and condensation into apoptotic bodies, which are subsequently cleared by phagocytosis. Autophagy-dependent cell death involves cytoplasmic vacuolization and the formation of autophagosomes, followed by lysosomal degradation^19^. Autophagy and the lysosomal system also play significant roles in cancer progression by promoting tumor growth and survival through the recycling of cellular components, and lysosomal enzymes have been identified as potential targets for cancer therapy^14,17,18^. Dysfunctional lysosomal transport has been linked to lysosomal storage disorders, neurodegenerative diseases, and cancer^14,17,18^. Hence, lysosomal transport is critical to cellular health.

These considerations regarding lysosomal transport inform the design of the present study as follows: When healthy cells exhibit normal lysosomal flux, Cdy6 dye binds to lysosomal vesicles in the healthy, live cells. If CLVs in live cells undergo cell death, CLVs in dead cells release their contents into the culture medium. Our method not only allows us to observe long-term real-time cell health under a microscope but also quantify it by performing an additional DMSO extraction step and measuring the resulting extract with a spectrometer. Several other studies have also targeted lysosome transport in cells to investigate and develop new methods for assessing cell viability and proliferation. For example, autophagy assays may monitor the turnover rate of the autophagosomal protein ATG8 and its homologs such as LC3 and GABARAP family proteins for biological discovery and therapeutic development^20^. Another study surveyed methods for the quantification of lysosomal membrane permeabilization, which is characteristic of lysosomal cell death^21^. Therefore, a simple and rapid assay of CLVs may serve as a useful method for quantifying and validating cell health in long-term real-time experiments conducted 2D and 3D in vitro models.

Meanwhile, 3D in vitro cell culture models have gained significance as a crucial tool for drug screening due to its ability to imitate the in vivo environment more effectively than conventional 2D models^22^. 3D in vitro cell culture models enable cell–cell interactions and interaction with the extracellular matrix, leading to more realistic cell behavior and drug responses^11,22^. 3D in vitro cell culture drug screening is performed with either scaffold-based and scaffold-free methods^11,22^. Scaffold-based methods use 3D scaffolds, such as collagen, alginate, or PEG, as a structure for cells to attach and grow on^13^. Scaffold-free methods, on the other hand, use hanging drop culture, microfluidics, or spheroid formation techniques to create 3D structures without scaffolds^22^.

Viability assays, high-content imaging, and gene expression analysis are some of the most commonly applied techniques for screening drugs using 3D in vitro cell cultures. More specifically, viability assays such as MTT and Alamar Blue are employed to assess the effects of drugs on cell proliferation and survival in 3D in vitro cell culture models^4,8,12,23^. High-content imaging enables the visualization of cell behavior and morphology in 3D, providing information on cell migration, invasion, and differentiation^8^. Gene expression analysis is employed to examine changes in gene expression patterns in response to drug treatment^24^. However, it can be difficult to accurately assess cell behavior and drug response through imaging and analysis of 3D structures. 3D microenvironment complexity and variability can also pose challenges to quantitative analysis. In particular, 3D in vitro cell culture systems have lower throughput compared to 2D cell cultures, which can limit the number of compounds that can be screened and curb the speed of drug discovery^11^. Furthermore, the availability and cost of 3D in vitro cell culture systems can be barriers to drug screening studies^11^. In addition, the lack of standardized protocols and guidelines for 3D in vitro cell culture-based drug screening can lead to inconsistent and unreliable results. Overall, assessing cell viability in 3D in vitro cell culture models faces several challenges, including complexity and long-term incubation, time and cost constraints, the need for suitable evaluation metrics, and complex data analysis.

To address these challenges, we explored a novel method using CLVs that specifically target lysosomal vesicles in live cells for application in 3D in vitro cell culture models such as 3D artificial skin models. In order to apply the CLV method to 3D in vitro cell culture models, we constructed a 3D dermal layer-on-frame assembled with a PES membrane, a collagen-CDy6 mixed hydrogel, and the CCD-986sk cell line, serving as an alternative skin model^25^. 3D artificial skin models are essential tools for testing without the use of animals and are employed to assess the safety and efficacy of cosmetics, pharmaceuticals, chemicals, and other products^25,26^. Furthermore, some 3D artificial skin models (EpiDerm, LabSkin Creations, MatTeck’s EpidermFT) are already commercially available. As our findings suggest, a simple and rapid CLV-mediated assay could be expected to make a significant contribution to assessing the health of cells in 3D artificial skin models, compensating for the current limitations in assay measurements of cell viability and cell proliferation in 2D and 3D in vitro cell culture models.

## Conclusion

In summary, this study describes a method for isolating and measuring CLVs from live cells using DMSO extraction and spectrometry to assess cell viability. A CLV-mediated assay is a simple and rapid method that can be used to assess cell status using both absorbance analysis and live cell imaging in 2D and 3D in vitro cell culture models under long-term and real-time conditions. This method provides a valuable tool for real-time monitoring of lysosomal transport in live cells using absorbance analysis and confocal microscopy.

## Materials and methods

### Reagents and standard 2D cell culture

The mitotic tracker probe CDy6, MTT, and DMSO (dimethyl sulfoxide) were purchased from Sigma (St. Louis, MO, USA). MS Collagen (Type 1 atelo-collagen from porcine skin) was obtained from MSBio, Inc. (Gyeonggi, Korea). Antibodies recognizing apoptosis western blot cocktail (pro/p17-caspase-3, cleaved PARP1, muscle actin) (ab136812) was obtained from Abcam (Cambridge, UK). Antibody recognizing actin (SC-58673) were obtained from Santa Cruz Biotechnology, Inc. (Santa Cruz, CA, USA). The HaCaT keratinocytes were purchased from CLS company (Germany). The human fibroblast cell line CCD-986sk cells were obtained from the Korea Cell Line Bank (KCLB, Seoul, Korea). The HaCaT keratinocytes and CCD-986sk cells were separately cultured in complete medium (DMEM-high glucose supplemented with 10% FBS and 1% PS (penicillin–streptomycin)). A stock solution of CDy6 was diluted in 100% DMSO at a concentration of 5 mg/mL and then diluted with complete medium to a final concentration of 100 ng/mL.

### A simple and rapid protocol using CLVs

To characterize the content and localization of CLVs in live cells in both 2D and 3D in vitro cell culture models, we developed a simple and rapid assay consisting of two parts: (1) the absorbance analysis of CLV-DMSO extract from CDy6-stained live cells using a spectrometer and (2) live cell imaging of CLVs using a confocal microscope. To perform the CLV absorbance analysis, cells were seeded in a 96-well black and white plate and cultured for 24 h prior to staining with CDy6. CDy6 (100 ng/mL) was added to the culture media and incubated at the time points indicated in Figs. 1, 2, 3, 4, 5, 6 and 7. To analyze the absorbance of the CLV-DMSO extract from CDy6-stained live cells, the cultured media were discarded, and 100 μL of 100% DMSO was added to each well. After 1 h at RT with gentle shaking on an orbital shaker, the CLV-DMSO extract was transferred to a new 96-well plate, and the fluorescence excitation and emission spectra were measured using a Spectra Max M2 plate reader. To assess the qualitative imaging analysis results made possible by the CLVs, fluorescent live-cell images were acquired using an Olympus FV1200 confocal microscope with 559 nm laser lines, a 10 × or 20 × objective lens, and a 60 × oil objective lens. To investigate the effects of CLVs on a 3D in vitro cell culture model, we generated a uniform and stable 3D dermal layer using the extrusion printing method with a 3DX Printer (T&R Biofab Co. Ltd, Siheung, Korea)^12,23^. To generate the 3D dermal layer, a 3% collagen solution was mixed with CDy6 (100 ng/mL), and CCD-986sk cells were mixed with the collagen-CDy6 hydrogel for fabrication. After gelation, the 3D dermal layer-on-frame constructions with polyether sulfone (PES) membrane filters (circle type, pore size 0.45 μm) were cultured in growth medium at 37 °C in a 5% CO_2_ incubator for the time points indicated in Figs. 6 and 7^25^. Calcein AM was used as a positive control for imaging and absorbance analysis of the live cells. The final concentration of calcein AM was 100 nM and all the experimental conditions for calcein AM were the same as for the CDy6 dye.

### Cell viability and cell proliferation assay

Cell viability and proliferation assays were prepared and performed using crystal violet staining and an MTT assay, as described previously^12,23^. Absorbance was determined for each dye using a spectrometer (Emax; Molecular Devices, Sunnyvale, CA, USA).

### Western blot analysis

The samples were disrupted using the homogenizer after which an ice-cold PRP-PREP protein extraction solution with a protease inhibitor cocktail (iNtRON Biotechnology, Inc, Seoul, Korea) was added, and the samples were homogenized by stainless steel beads (Qiagen, Cam USA). Protein concentration was assessed using a BCA-kit (Thermo Scientific, Rockford, IL, USA). An equal amount of protein (50 µg) from each sample was loaded onto 10% to 12% SDS gel, and transferred to a PVDF membrane (Merk Millipore, MA, USA). The membranes were blocked for 2 h at RT with 5% nonfat dry milk in PBS containing 0.1% Tween-20, and incubated with anti-bodies (1:1000) overnight at 4 °C (Supplementary Table 1). After washing three times, the membranes were incubated with a horseradish peroxidase-conjugated secondary antibody (1:5000) at RT for 2 h and visualized with a chemiluminescence substrate.

### Statistical analysis

Student’s *t-*tests (for comparisons of two groups) or a one-way analysis of variance (ANOVA) (for comparisons of three or more groups) followed by Tukey post hoc tests were used for the statistical analyses. SPSS software ver. 17.0 (SPSS, Chicago, IL) was used. A value of *P* < 0.05 was considered significant. Data are expressed as means ± standard error of the mean (SEM). Data analysis was carried out using GraphPad Prism software (GraphPad Software Inc). *P < 0.05–0.01, **P < 0.01–0.001, and ***, $$$*P* < 0.001 vs. corresponding controls. All error bars represent the standard deviation of three or more biological replicates.

### Supplementary Information

Supplementary Movie S1.

Supplementary Movie S2.

Supplementary Information.

## Data Availability

All data generated or analyzed during this study are included in this published article [and its supplementary information files]. And all data that support the findings of the present study are available from the corresponding authors (phdjeongym12@tukorea.ac.kr) upon reasonable request.
