# Supplementary material for: A simple and rapid assay of lysosomal-targeting CDy6 for long-term real-time viability assessments in 2D and 3D in vitro culture models
Source: Sci Rep. 2023 Dec 27;13:23038. doi: 10.1038/s41598-023-49844-1 (PMC10754854; doi:10.1038/s41598-023-49844-1)
Supplement: Supplementary file 3 — Supplementary Information. [file 41598_2023_49844_MOESM3_ESM.docx]

**A simple and rapid assay of lysosomal-targeting CDy6 for long-term real-time viability assessments in 2D and 3D *in vitro* culture models.**

Chanhan Kang, Won-Soo Yun, Yun-Mi Jeong*

Department of Mechanical Engineering, Tech University of Korea, 237 Sangidaehak street, Si-heung city, Republic of Korea.

*Corresponding author: phdjeongym12@tukorea.ac.kr

**Method**

**Cell culture**

RPE1 cells (hTERT retinal pigmented epithelial cell line, American Type Culture Collection [ATCC] strain CRL-4000) were maintained in ATCC-formulated DMEM F12 1:1 supplemented with 10% FBS with 0.01 mg/mL hygromycin B (Life Technologies). The human osteosarcoma U2OS cells were maintained in DMEM high glucose supplemented with 8% fetal bovine serum (FBS)/1% penicillin and streptomycin (PS) at 37 °C with 5% CO_2_.

**Time-Lapse Imaging**

To obtain long-term, time-lapsed images of CLVs, we used a Nikon Biostation IM system as previously described^10^. Cells were seeded onto a 35 mm dish, stained with CDy6 (0.1 μM) in complete medium, and placed in a Nikon Biostation IM system (epifluorescence lamp, 6%; exposure time, 1/10 s; interval time, every 3 min for 50–72 h at 37 °C with 5% CO_2_). Ten experiments were performed. A minimum of 200 cells was monitored per experiment. Relative fluorescence intensity in CDy6 was visualized using NIS-Elements 3.10 software.

**Absorbance measurement of CDy6**

A stock solution of CDy6 were first prepared in PBS buffer at a concentration of 0.1 mM, then diluted with DMSO to a final concentration of 100 nM. The fluorescence excitation and emission spectra were measured using a Spectra Max M2 plate reader (Molecular Devices Corp, USA).


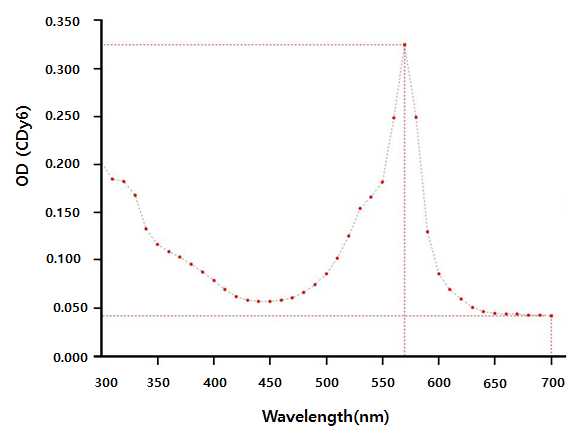
**Fig. S1. Normalized absorption of CDy6.**

**
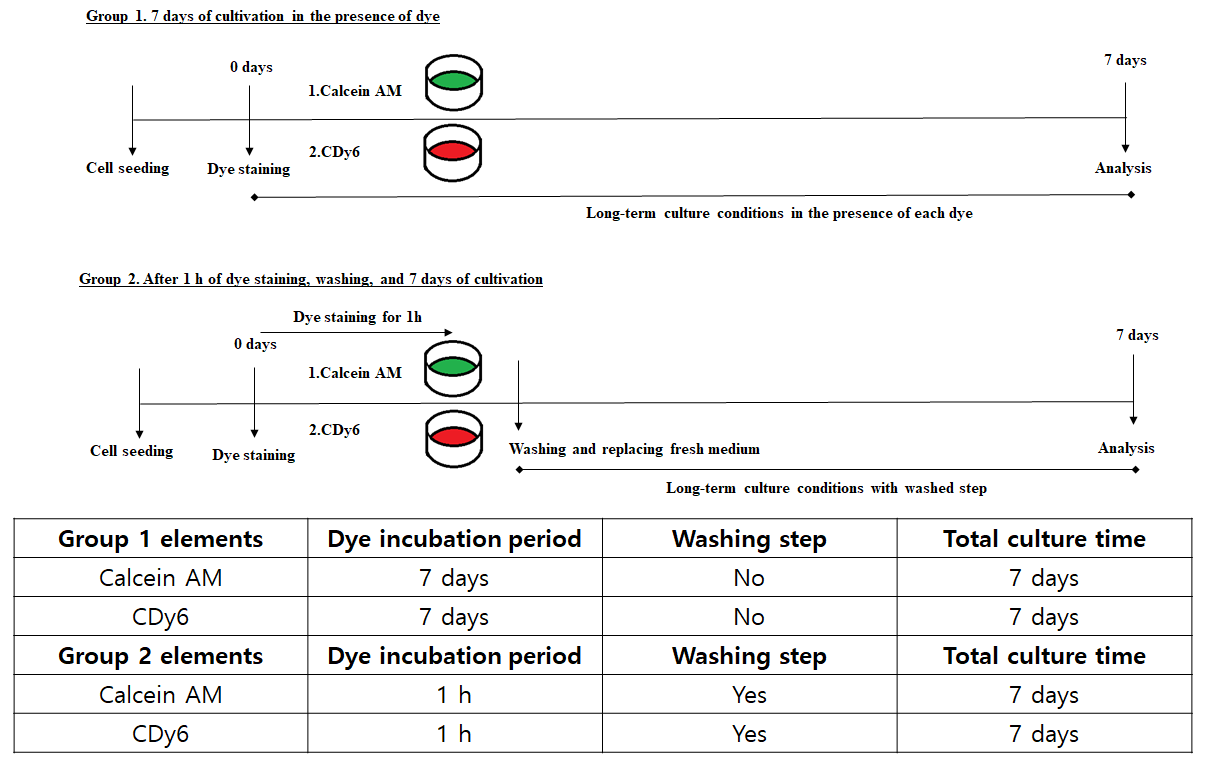
Fig. S2. Related to Figure 3. The experimental design for two groups: 1) 7 days of cultivation in the presence of each dye. 2) After 1 h of each dye staining, followed by washing and 7 days of cultivation.** The difference between the two groups becomes apparent after 1 h of staining, washing, and 7 days of cultivation. The first group (group 1) was cultured for 7 days in the presence of each dye in the medium, while the second group (group 2) underwent dye staining for 1 h, then was washed and replenished with fresh medium for 7 days of cultivation. The table provides a summary of the experimental setup related to Fig. 3.

**Movie S1. Related to Figure 1. The behavior of CLVs in U2OS in the short-term real-time condition (24 h).**

**Movie S2. Related to Figure 5. The behavior of CLVs in RPE1 in the long-term real-time condition (92 h).**

Table 1. Validated primary antibodies for western blot analysis.

| Antibody | Dilution | Solvent | Supplier | Molecular Weight |
| --- | --- | --- | --- | --- |
| actin | 1:5000 | 5% BSA in TBST | Santa Cruz | 42 kDa |
| apoptosis western blot cocktail | 1:1000 | 5% BSA in TBST | Abcam | Cleaved-PARP, 89 kDa; procaspase-3, 32 kDa; actin, 42 kDa |


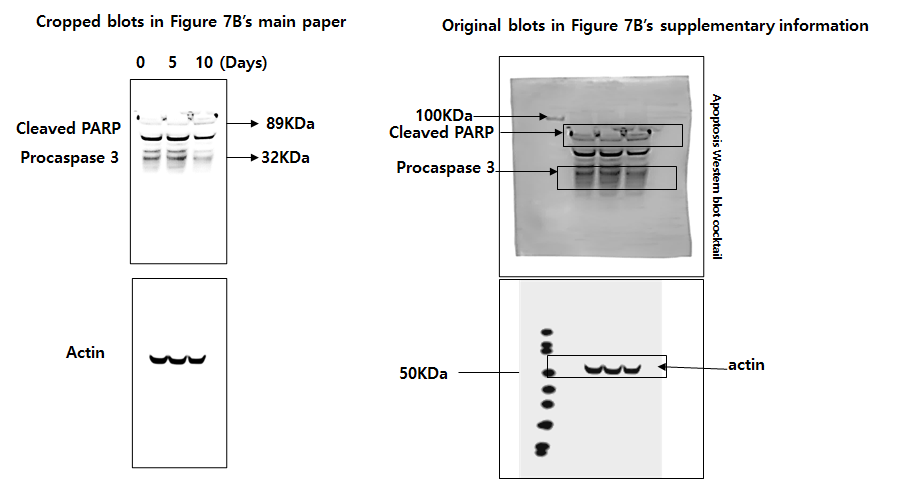


Fig. 7B. Raw data of western blot analysis.
